# MDSuite: comprehensive post-processing tool for particle simulations

**DOI:** 10.1186/s13321-023-00687-y

**Published:** 2023-02-11

**Authors:** Samuel Tovey, Fabian Zills, Francisco Torres-Herrador, Christoph Lohrmann, Marco Brückner, Christian Holm

**Affiliations:** 1 Institute for Computational Physics, Universität Stuttgart, Stuttgart, Germany; 2 Aeronautics and Aerospace Department, von Karman Institute for Fluid Dynamics, Rhode-St-Genese, Belgium; 3 Thermo and Fluid Dynamics (FLOW), Vrije Universiteit Brussel, Brussels, Belgium; 4 Laboratory for Chemical Technology (LCT), Ghent University, Ghent, Belgium

**Keywords:** Molecular dynamics, Computational physics, Material properties, High performance computing, TensorFlow, FAIR data

## Abstract

Particle-Based (PB) simulations, including Molecular Dynamics (MD), provide access to system observables that are not easily available experimentally. However, in most cases, PB data needs to be processed after a simulation to extract these observables. One of the main challenges in post-processing PB simulations is managing the large amounts of data typically generated without incurring memory or computational capacity limitations. In this work, we introduce the post-processing tool: MDSuite. This software, developed in Python, combines state-of-the-art computing technologies such as TensorFlow, with modern data management tools such as HDF5 and SQL for a fast, scalable, and accurate PB data processing engine. This package, built around the principles of FAIR data, provides a memory safe, parallelized, and GPU accelerated environment for the analysis of particle simulations. The software currently offers 17 calculators for the computation of properties including diffusion coefficients, thermal conductivity, viscosity, radial distribution functions, coordination numbers, and more. Further, the object-oriented framework allows for the rapid implementation of new calculators or file-readers for different simulation software. The Python front-end provides a familiar interface for many users in the scientific community and a mild learning curve for the inexperienced. Future developments will include the introduction of more analysis associated with ab-initio methods, colloidal/macroscopic particle methods, and extension to experimental data.

**Supplementary Information:**

The online version contains supplementary material available at 10.1186/s13321-023-00687-y.

## Introduction

Particle-based (PB) simulations, of which we declare Molecular dynamics (MD) to be a subset, are a cornerstone of modern computational physics, chemistry, biology, and engineering. The benefit of PB simulations comes in their access to system observables and properties that are otherwise unavailable experimentally. Whilst the method itself involves the sampling of configuration space under constraints imposed by defined interactions, it is the analysis of these configurations that leads to insights in medicine [1–5], battery technology [6–15], astrophysics [16], materials engineering [17–21], and much more. When running simulations, one is faced with the choice of either performing On-The-Fly (OTF) analysis, or post-processing their simulation data to extract information. In the case of OTF analysis, it is often difficult to know beforehand parameters such as measurement range, number of samples, or bin resolution, that will be required in the calculations. For this reason, post-processing of simulation results has become a preferred solution, particularly in cases where simulations are computationally expensive. Another important consideration is the number of simulations that will be studied. In the case of temperature or al-chemical comparison, often several large simulations will be run under varying conditions. In these cases, it is convenient to have access to post-processing tools that can track and analyse each simulation as well as compare the results of the analysis. Several post-processing tools have been developed in the past and are used by scientists around the world including MDAnalysis [22], PyLAT [23], pyLOOS, mdtraj [24], pytraj [25], freud [26], and VMD [27] to name some of the most popular. These programs, each having their own strengths, follow a similar workflow:

1. Load a trajectory object
2. Perform analysis
3. Close trajectory object

Whilst this method meets the demands of many users in the PB community, it is positioned more as a tool for the analysis of single simulations and less as an integral part of the scientific process. In the direction of managing many simulations, there also exists several data management tools including datreant [28] and signac [29]. These packages allow scientists to build large data workflows and track their states as they use other libraries to perform analysis. Put together, the post-processing libraries as well as data management packages can assist scientists in handling their simulations so long as interoperability is well handled by the users. A downside of course is that the post processing and the experiment handling is separated which can result in a more complex interface and reduced usability.

In this paper we introduce MDSuite, a post-processing program for PB simulations that allows for the collection and analysis of simulation data from many simulations under a common framework. MDSuite differs from the previously mentioned programs in that it takes raw simulation data and generates a new database more suited for rapid post-processing. This database structure, hereafter referred to as MDS-DB, allows data to be loaded only when it is pertinent to the analysis being performed. Furthermore, the structure is such that properties of the particles (i.e. positions, stress, and forces) are separated by species at the time of database construction, thereby eliminating expensive computational overhead during analysis. Furthermore, MDSuite uses an enveloping object that we have named the Project class, in which the results of any number of simulations may be contained, analysed, and compared to one another.

The proceeding work is structured as follows. First we discuss the guiding principles of the MDSuite Python library, introducing the Project and Experiment class objects as well as how they work together to create an optimised and user-friendly analysis environment. This is followed by a discussion into the MDS-DB structure used by MDSuite to store simulation data. We then introduce the different properties that can currently be computed using the MDSuite code including information regarding the implementation of the analysis. After the introduction of the calculators, a brief performance review is presented in order to outline how the MDSuite code has been written to take full advantage of the latest high performance computing systems. Finally, we introduce the outlook of the MDSuite package including the extension beyond simulations as well as general performance optimization.

## Implementation

### Software architecture

The governing principle behind MDSuite is the simplification of PB investigations. MDSuite approaches this by providing an infrastructure in which simulation data and analysis results from any number of simulations may be stored, (re-)analyzed, and compared.

**Fig. 1:**
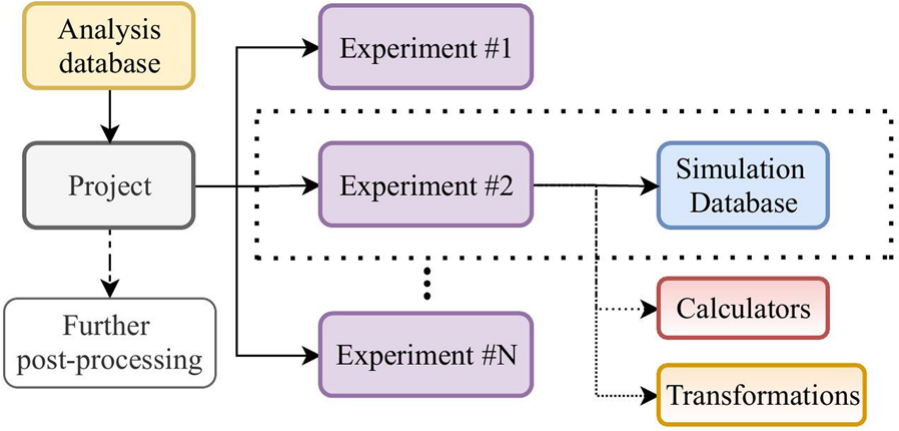
Structure of a MDSuite project. Each experiment stores data in its own database and has access to the Calculators and Transformations

The two integral components of this infrastructure are the Project class and the Experiment class (Fig. 1).

The Experiment class contains all the information pertaining to a single simulation including particle trajectories, net system properties such as fluxes, and thermodynamic information such as temperature and pressure. This data is stored in a compressed manner within the MDS-DB. Simulation data can be added to the Experiment at one time or iteratively over the course of a simulation as might be necessary in the case where several runs are required. Further to the storage of trajectory data, the Experiment class connects to the MDSuite calculators ("Calculators" section) which are used to perform analysis. The results of the analyses, including any series data generated in the process, are also stored by the Experiment class in an SQL database (SQL-DB). This means that once an analysis has been performed, the raw data such as an autocorrelation function, or a radial distribution function, as well as the outcome of the analysis such as a diffusion coefficient or coordination number, may be accessed at any time without needing to repeat the calculations. Furthermore, the use of an SQL database structure allows for the storage of the parameters used in the calculation such as data range, correlation time, or bin spacing. This means that analyzed data can be queried from a database and compared along with the parameters of the calculation. One may also use this feature for further processing such the common practice of fitting the autocorrelation function with double exponentials for the determination of the thermal conductivity with all necessary information about the computation stored. An additional important feature of the trajectory data stored by the Experiment class is the assigned version. If a computation is performed with a fixed set of parameters before additional trajectory data is added, it is important that when this computation is called again after the addition of data, that it is recomputed and not simply loaded from the database. This is handled by a version system that assigns a new number to each state of the MDS-DB as trajectory data is added. If new data is parsed into the database, the version will be updated and the computations performed on the old set of data will become stale such that computations with identical parameters may be re-performed.

The Project class acts as a container for multiple Experiment objects and can be used to collect property values for each of the classes and compare them with respect to a desired parameter. Experiments can be added and removed from the Project class at any time during an investigation and there is no need to know beforehand how many will be performed. Fundamental simulation properties such as thermostat temperature, time-step, and species information are stored in the SQL-DB for each experiment. Due to the internal use of SI units in MDSuite, simulations performed using different systems of units or simulation engines can still be directly compared with one another. As an example, consider the comparisons of two inter-atomic potentials, each constructed with a specific unit system in mind. Utilizing the MDSuite framework eliminates the necessity of unit conversion whilst also providing a simple means for comparing analysis results.

#### SQL-DB

As was mentioned earlier, MDSuite utilizes an SQL database for the management of simulation parameter data and results of computations. This database is built using the SQLAlchemy package [30] which allows for multiple back-end SQL engines. Data stored within the database can either be series type data, e.g. an autocorrelation function, or the results of some analysis such as diffusion coefficients along with the uncertainty associated with this measurement. This data will also have associated with it meta-data including computation range, correlation time, and number of configurations. This type of record keeping is essential in the communication of results in case of publication as is in line with the FAIR data principles [31]. FAIR data guidelines are a framework for improving the way scientists approach the Findability, Accessibility, Interoperability, and Reuse of digital assets [31]. In MDSuite, the storage of all computation results along with their parameters provides an environment for such a framework as these results are freely accessible from outside MDSuite, they can be hosted online, they will provide data in standard data types, and provide a complete parameter set describing how the data was produced. Utilization of the SQL-DB not only allows for simple navigation of data through the Project class, but also for the use of generic SQL database navigation tools for studying data graphically.

#### MDS-DB

**Fig. 2:**
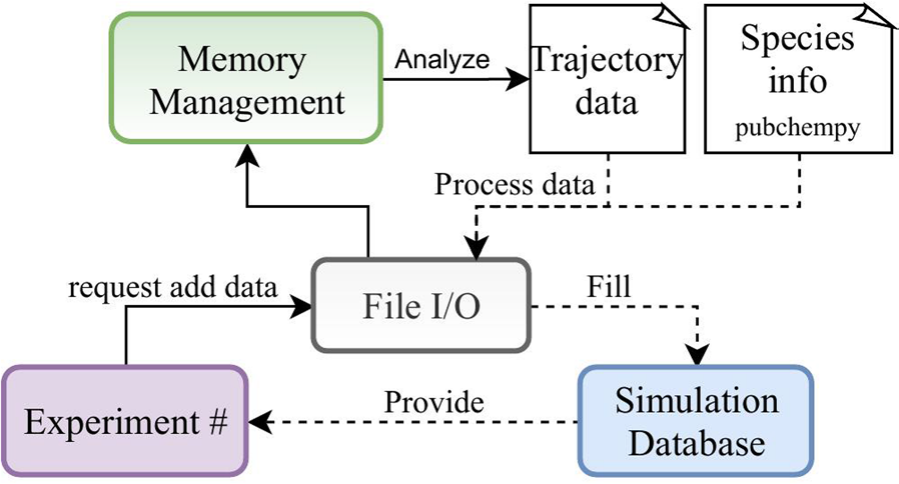
Database generation logic. Solid lines indicate actions and dashed lines data flow

The database generation process is one of the unique features of MDSuite. Typically, MD codes dump the results of simulations into data files of varying format which may contain trajectory information of the particles or net system information such as fluxes and temperatures. In order to avoid complications with calculators, MDSuite reads simulation data and constructs a database based upon the HDF5 hierarchical data format [32]. Currently, MDSuite uses a slight variation of the established H5MD database structure [33], however, this will change in the near future and be brought completely inline with this standard. The database construction process is depicted in Fig. 2. When data is added to an Experiment, the File I/O module will choose the corresponding reader with which to process the data. Simultaneously, the Memory Management module will analyze the original trajectory file and decide the appropriate batching size to process the data. This allows one to efficiently parse large datasets on a machine with limited memory. As the batches of data are read, they are stored in the simulation database. Additionally, the PubChemPy [34] library is called to expand species information to real element specific data such as mass and charge which is then stored in the SQL-DB.

**Fig. 3:**
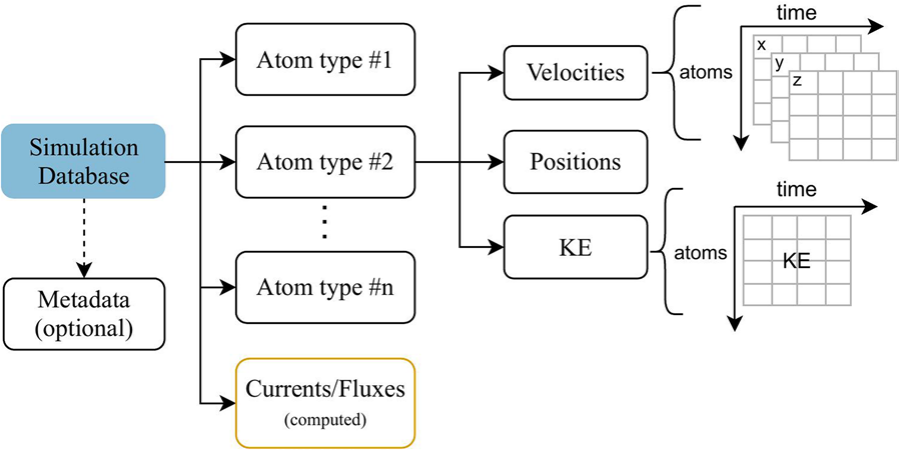
Typical structure of the simulation database. For each atom type, sub-groups for each variable are created. Data is then stored in tables. Currents or fluxes are also stored as global quantities

The MDS-DB, rather than trivially storing configurations of atoms, splits the information into groups and sub-groups by property (Fig. 3). As an example, in the generation of an MDS-DB for a system of NaCl, positions, forces, and velocities for all sodium and chloride atoms in the simulation will be split first into the groups Na and Cl and then into sub-groups of positions, velocities, and forces. This extends naturally to the use of coarse-grained descriptions where molecules are used as the groups and center of mass positions and velocities are stored. In the case of system wide properties such as global flux (eg. thermal flux), data is simply stored in a group with the name of the flux. Currents, fluxes, or other global properties can also be computed inside MDSuite using the transformations and stored in the MDS-DB. With this structure, any simulation data may be stored in a simple, compressed format. The motivation behind such a partitioning scheme is rooted in programming simplicity, memory safety, and improved computational performance. Programming simplicity becomes evident when looking at calculator definitions. In MDSuite, each calculator has a set of properties that it requires to perform the calculation. With a partitioned database scheme this becomes universal across file formats and makes writing new calculators more efficient. Furthermore, with species-wise separation it becomes simple to perform computations on only a selection of species in a simulation. Beyond simplicity in writing calculators, memory safety also becomes easier to handle with data being split by property. This is because when MDSuite is called to compute a diffusion coefficient for a single species, it can load only the specific positions of the single species rather than having to search through all trajectory data and filter it for the desired components. In this way, memory usage calculations can be performed on a well-defined amount of data. Finally, while using a partitioning scheme may increase the initial processing time when creating an experiment, it means that data is read from a file precisely once and during analysis there is no need for searching through trajectory data.

As different MD simulation engines have different output formats, MDSuite implements readers to pre-process simulation data and prepare it for storage. MDSuite utilizes the Chemfiles library [35] as a foundation for file-reading thereby giving access to over 21 different formats including those for large simulations engines such as GROMACS [36], AMBER [37], CHARMM [38] and LAMMPS [39, 40]. Furthermore, the MDSuite file readers are written such that it is simple to add new readers for custom simulation outputs. This has been used, for example, to store the output of an ESPResSo [41] simulation of a bacteria study, initially stored as a pandas [42, 43] DataFrame

#### User interface

The aim of the MDSuite user interface is to allow for easy and understandable communication with the Calculators and Transformations. To this end, MDSuite is distributed as a Python package with a streamlined API. That is to say, users typically require only a single package import and only access methods from a single class. To aid in usability, many user-focused methods have been added to perform simple tasks such as accessing stored data, changing names of MDS database groups, and updating physical properties of species. These features keep the focus on scientific work rather than computational baggage. This usability is particularly noticeable when using the package through Jupyter notebooks [44, 45], as this allows direct interaction with results as they are calculated.

Code sample 1 demonstrates the MDSuite API for the analysis of two molten salt systems of NaCl and LiCl.
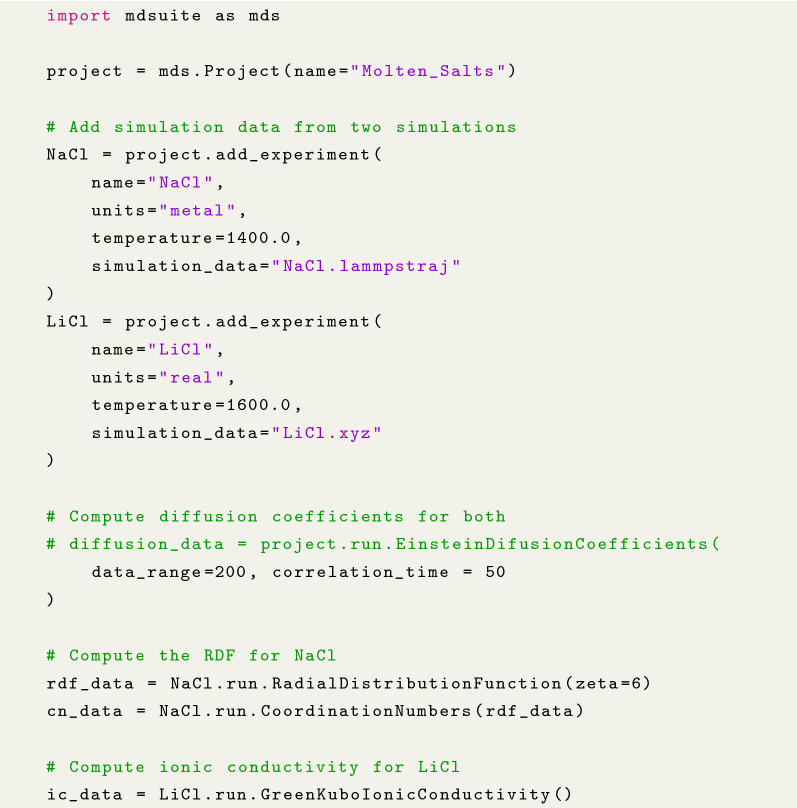


**Code sample 1** Example of MDSuite API for the analysis of two molten salts. In this case, self-diffusion coefficients are computed for both salts whereas the radial distribution functions and ionic conductivitiy are computed only for the NaCl and LiCl respectively.

### Calculators

A driving factor in the design logic of MDSuite was the desire to be able to perform a multitude of analyses on a simulation trajectory under a single framework. The construction of the MDS-DB allows for data to be rapidly loaded based on the particle type and property under consideration. With this approach, a number of methods for analysis become possible in a fast and memory safe manner. This section discusses the different analysis options available to the MDSuite user with some details about their implementation.

All analyses in MDSuite are built from the Calculator parent class. Every other calculator inherits from this class and implements the actual computation. The benefit of this is that the Experiment class is agnostic towards the inner workings of calculator objects and interfaces with them in the same manner, regardless of the computation being performed. Furthermore, by structuring the calculator implementations as children of a parent class, additional calculators may be easily added by users.

**Fig. 4:**
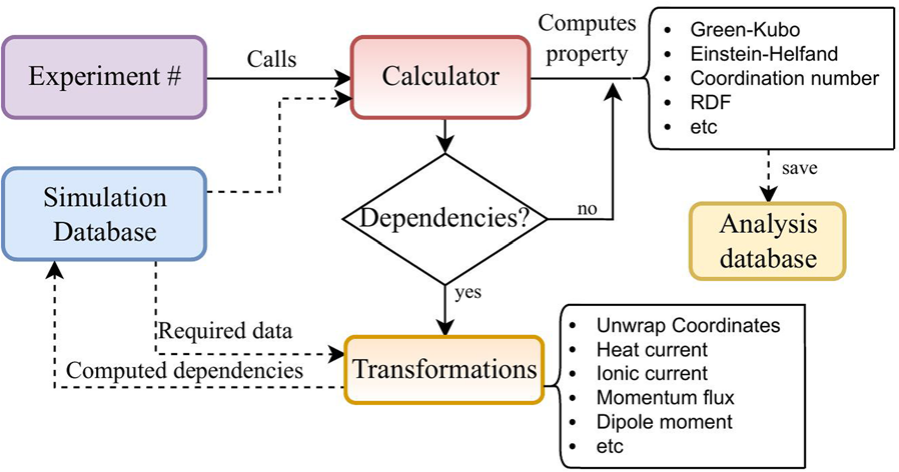
Execution logic of a Calculator. Continuous lines depict actions, dashed lines depict data flow

The execution logic of a calculator is depicted in Fig. 4. When a Project requests that a particular calculation be performed, the corresponding Calculator will be called. This Calculator will request the required data from the MDS-DB ("MDS-DB" section). If this data is not available, the Calculator will make use of the appropriate Transformation to compute it. The computed data will be stored in the MDS-DB to avoid re-computation in the event that the analysis is repeated. Once all the dependencies are available, the calculation of the property will proceed. Finally, the results will be stored in the SQL-DB.

All calculators in MDSuite may be called simply from the Project class with:
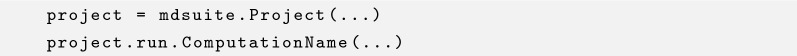


In order to optimize the performance of the calculators, as well as to take advantage of modern hardware, MDSuite utilizes the TensorFlow Python library [46] to allow not only for optimized tensor calculations, but also for immediate deployment on GPU and parallel processing with a small increase in import time due to device registration. TensorFlow is also used in the construction of optimized data pipelines for the computations where TensorFlow Datasets, used for elements of batching, are employed. Memory safety is handled both with configuration-wise batching and atom-wise mini-batching if necessary (see Additional file 1). The performance improvements of the TensorFlow library are discussed in more detail in "Performance" section where strong-scaling tests have been performed.

The remainder of this section will discuss the available calculators.

### Structural properties

The computation of structural properties in MDSuite largely revolves around the radial distribution function. The following section outlines the various structural analyses available to users as well as what each of them describes.

#### Radial distribution function

Radial distribution functions (RDFs) describe the particle density of species $\alpha$ at a distance *r* from a particle of species $\beta$ [47]. This may be calculated directly from the positions of particles within a simulation by:

1 $$\begin{aligned} g_{\alpha \beta }(r) = \frac{N_{\alpha }N_{\beta }}{V} \sum _{i=1}^{N_\alpha } \sum _{j=1}^{N_\beta } \langle \delta (r - |\textbf{r}^{\alpha }_i - \textbf{r}^{\beta }_j|) \rangle , \end{aligned}$$

where $g_{\alpha \beta }$ is the RDF of species $\alpha$ with respect to species $\beta$, $N_{\alpha }$ is the number of particles of species $\alpha$, *V* is the box volume, and $\textbf{r}_{i}$ is the position of particle i.

#### Angular distribution function

Angular distribution functions (ADFs), similar to the RDF, describe the distribution of the angle between three atoms. ADFs can be used to better understand bonding between specific species as well as to identify breakdown in structure under changing environments. An ADF is computed with reference to three atoms in the system and is calculated by:

2 $$\begin{aligned} g_{\alpha \beta \gamma } (\theta ) = \frac{1}{N_{\alpha }N_{\beta }N_{\gamma }}\sum _{i j k \in \alpha \beta \gamma }\frac{1}{|r_{i j}|^{\zeta } |r_{i k}|^{\zeta }} \langle \delta (\theta - \theta _{i j k}) \rangle , \end{aligned}$$

where the angle being measured is between the triangle formed with the atom of species $\alpha$ at the centre and $\zeta$ is a sensitivity factor used to highlight peaks and relevant distances. This sensitivity index can be useful in filtering out angles occurring at large distances from the central particle. This is because, during the ADF calculation, no information about distance between the particles is used in the calculation of the angles.

#### Coordination numbers

Coordination numbers describe the number of particles surrounding a reference particle at a chosen distance and are related to the ordering in the system [48]. In the case of a crystalline structure, this reduces simply to the number of particles bonded to the reference particle [49]. In a liquid system this becomes a less rigorous definition and the coordination numbers are often calculated at different points along the RDF referred to as coordination shells [48]. MDSuite uses the definition of coordination numbers taken from Waseda [48]:

3 $$\begin{aligned} n_{\alpha \beta }^{i} = 4 \pi \rho \int \limits _{r_{i-1}}^{r_{i}} \textrm{d}r r^{2} g_{\alpha \beta }(r), \end{aligned}$$

where $i \ge 1$ is the coordination number to be calculated associated with a chosen coordination shell. The integration range is taken at minimums between successive peaks in the RDF. MDSuite uses the Golden-Section search algorithm [50] along with a Savitzky-Golay [51] filter to identify the minimums in the RDFs and perform the analysis correctly (see Additional file 1). The parameters of the filter are tunable at runtime.

#### Potential of mean-force

Potential of mean-force (PMF) is a measurement of how the free energy of a system changes as a function of inter-atomic distance and may be calculated directly from the RDF as [52, 53]:

4 $$\begin{aligned} w_{\alpha \beta }(r) = -k_{\text {B}}T \ln g_{\alpha \beta }(r). \end{aligned}$$

The PMF ($w_{\alpha \beta }$), computed between species $\alpha$ and $\beta$, can be used to understand effective binding strength between atomic species [14]. MDSuite utilizes the minima finding algorithm discussed for the coordination numbers to identify the minimum value of the PMF.

#### Kirkwood-Buff integrals

Kirkwood-Buff (KB) integrals are a fundamental component of the Kirkwood-Buff solution theory developed by John G. Kirkwood and Frank P. Buff aimed at relating microscopic properties of a solution to its thermodynamic quantities [54]. The integrals are calculated directly from an RDF by:

5 $$\begin{aligned} G_{\alpha \beta } = 4\pi \int \limits _{0}^{\infty }\textrm{d}r r^{2}(g_{\alpha \beta }(r) - 1). \end{aligned}$$

The KB integral ($G_{\alpha \beta }$), computed between species $\alpha$ and $\beta$, can be used to better understand preferential binding between species in a system [55].

### Dynamic properties

Dynamic calculations are those that take place with respect to time. As such, there exists correlation between properties of particles and therefore, species attention must be paid when performing computations. In all dynamic calculations in MDSuite, ensembling is performed to ensure correct statistics [56]. This ensembling is represented by the angled brackets in each of the calculations to follow. In each computation, the user may set a desired correlation time to decide over what time range this ensembling occurs and thereby also computing correct errors.

Typically in particle simulations there are two approaches to the computation of dynamic properties, Green-Kubo, and Einstein-Helfand. MDSuite implements both of these calculation approaches and therefore, a brief discussion of each is included.

#### Green-Kubo calculations

**Fig. 5:**
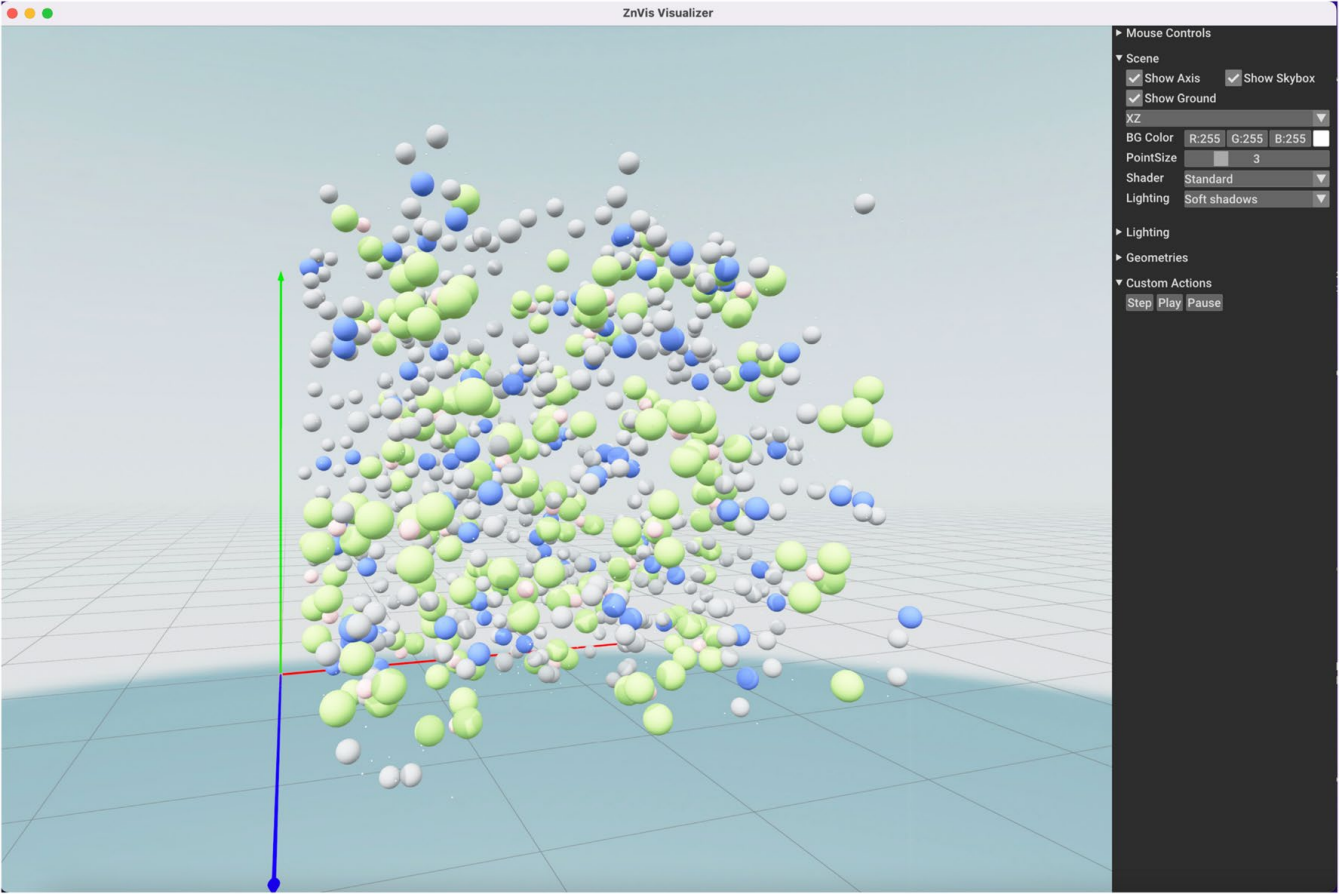
A snapshot from an MD simulation of the BMIM-BF4 ionic liquid

The first method discussed is the Green-Kubo approach [57–59] which uses autocorrelation functions to compute dynamic properties from a flux in the system by:

6 $$\begin{aligned} \lambda = \frac{\omega ^{\text {GK}}}{d\cdot V}\int \limits _{0}^{\infty }dt \langle \varvec{\eta }(t) \cdot \varvec{\eta }(0) \rangle , \end{aligned}$$

where $\omega ^{\text {GK}}$ is some calculation specific pre-factor, *d* is the dimension, and $\varvec{\eta }$ is the flux on which autocorrelation is computed. In reality, the integral cannot be taken to infinity and rather a cutoff value must be chosen at which to integrate the system. As this can take optimization, MDSuite allows the user to set an integration range and then computes the running integral, along with the uncertainty, on the function up to this value. This approach allows users to identify a converged value for the integral. This running integral can be seen in Fig. 5.

#### Einstein-Helfand computations

In the case of an Einstein-Helfand computation, rather than use an autocorrelation function we look at the gradient of a mean square displacement. The property for which we look at the mean square displacement is often the integral of the flux used in the corresponding Green-Kubo approach. In this case one would like to solve:

7 $$\begin{aligned} \lambda = \lim _{t \rightarrow \infty } \frac{\omega ^{\text {EH}}}{2\cdot d\cdot t}\langle |\varvec{\zeta }(t) - \varvec{\zeta }(0)|^{2} \rangle , \end{aligned}$$

where $\varvec{\zeta }$ is the integrated flux, $\omega ^{\text {EH}}$ is a property species pre-factor, and *d* is the dimension.

In the remainder of this section each dynamic calculator available in MDSuite is discussed.

#### Viscosity

Viscosity is a fluids resistance to deformation. In MDSuite, the viscosity can be calculated using both the Green-Kubo relation and the Einstein-Helfand relation [60]. Assuming isotropy, the equation to compute viscosity by means of Green-Kubo reads [60]:

8 $$\begin{aligned} \kappa = \frac{V}{3 k_\text {B} T} \int \limits _{0}^{\infty } \textrm{d}t \langle P_{a,b}(0) \cdot P_{a,b}(t) \rangle \qquad (a \ne b \in \{x,y,z\}) \end{aligned}$$

where *V* is the volume of the system, $k_\text {B}$ is the Boltzmann constant, *T* is the temperature, and $P_{a,b}(t)$ are the off-diagonal components of the stress tensor at time *t*. The Einstein-Helfand approach uses a limit observation to determine the viscosity [60] with:

9 $$\begin{aligned} \kappa = \frac{1}{V k_\text {B} T} \lim _{t \rightarrow \infty } \frac{1}{2t} \langle [L_{a,b}^i(t)-L_{a,b}^i(0)]^2 \rangle , \end{aligned}$$

where $L_{a,b}(t)$ is defined as:

10 $$\begin{aligned} L_{a,b}^i(t) = p_{a}^i(t) \cdot r^i_b(t), \end{aligned}$$

and where $p_{a}^i$ and $r_{b}(t)$ are the momentum and position of particle *i* respectively.

#### Self-diffusion coefficients

Self-diffusion coefficients describe the average rate at which atoms of species $\alpha$ diffuse through the bulk. They may be calculated from a Green-Kubo relation by:

11 $$\begin{aligned} D_{\alpha } = \frac{1}{3N_{\alpha }}\int \limits _{0}^{\infty } \textrm{d}t \sum \limits _{i}^{N_{\alpha }} \langle \textbf{v}^{\alpha }_{i}(t)\cdot \textbf{v}^{\alpha }_{i}(0) \rangle , \end{aligned}$$

where $\textbf{v}^{\alpha }_{i}$ is the velocity of particle *i* of species $\alpha$. MDSuite also provides the corresponding Einstein relation, written [47]:

12 $$\begin{aligned} D_{\alpha } = \frac{1}{2\cdot d\cdot t}\frac{1}{N_{\alpha }} \lim _{t \rightarrow \infty } \sum \limits _{i}^{N_{\alpha }}\langle |\textbf{r}_{i}^{\alpha }(t) - \textbf{r}_{i}^{\alpha }(0)|^{2} \rangle , \end{aligned}$$

where *d* is the dimension of the system.

#### Distinct-diffusion coefficients

A related property to the self-diffusion coefficients are the so-called distinct diffusion coefficients which provide information about atom correlation in a system [61]. The Green-Kubo relation implemented in MDSuite is written:

13 $$\begin{aligned} D_{\alpha \beta } = \frac{1}{3}\frac{1}{N_{\alpha }\cdot N_{\beta }}\int \limits _{0}^{\infty } \textrm{d}t \sum \limits _{i}^{N_{\alpha }}\sum \limits _{j}^{N_{\beta }} \langle \textbf{v}^{\alpha }_{i}(t)\cdot \textbf{v}^{\beta }_{j}(0) \rangle , \end{aligned}$$

where summation is performed over distinct atoms *i* and *j*. In the case of $\alpha = \beta$, the condition $i \ne j$ is enforced. The corresponding Einstein formulation is also implemented as:

14 $$\begin{aligned} D_{\alpha \beta } = \frac{1}{N_{\alpha }\cdot N_{\beta }}\frac{1}{2\cdot d\cdot t} \lim _{t \rightarrow \infty }&\sum \limits _{i}^{N_{\alpha }}\sum \limits _{j}^{N_{\beta }}\langle (\textbf{r}_{i}^{\alpha }(t) - \textbf{r}_{i}^{\alpha }(0))\cdot (\textbf{r}_{j}^{\beta }(t) - \textbf{r}_{j}^{\beta }(0)) \rangle . \end{aligned}$$

#### Ionic conductivity

The ionic conductivity of a system describes the ability of a material to dissipate electrical energy based on the mobility and charge of the constituent ions. Ionic conductivity may be calculated by the Green-Kubo relation as [62]:

15 $$\begin{aligned} \sigma = \frac{\beta }{3V}\int \limits _{0}^{\infty } \textrm{d}t \langle \textbf{J}^{\sigma }(t) \cdot \textbf{J}^{\sigma }(0) \rangle , \end{aligned}$$

where $\beta = \frac{1}{k_\text {B}T}$, V is the volume of the system, and $\textbf{J}^{\sigma } = q\sum \limits _{i}^{N}z_{i}\textbf{v}_{i}$ is the ionic current. An alternative approach, denoted the Einstein-Helfand method, involves studying the mean square displacement of the translational dipole moment as:

16 $$\begin{aligned} \sigma = \lim _{t \rightarrow \infty } \frac{\beta }{2\cdot d\cdot t} \langle |\textbf{M}(t) - \textbf{M}(0)|^{2} \rangle , \end{aligned}$$

where $\textbf{M} = q\sum \limits ^{N}_{i}z_{i}\textbf{r}_{i}$ is the translational dipole moment of the system. To further probe the effects of ion correlation in a system, MDSuite also calculates the Nernst-Einstein ionic conductivity which uses the self-diffusion coefficients to approximate the property as

17 $$\begin{aligned} \sigma ^{NE} = \frac{q^{2}\beta }{3V}\Bigg (\sum _{\alpha }x_{\alpha }z^{2}_{\alpha }D_{\alpha } \Bigg ), \end{aligned}$$

where $x_{\alpha }$ is the mass fraction of species $\alpha$, $z_{\alpha }$ is the ion charge, and $D_{\alpha }$ is the self-diffusion coefficient. The Nernst-Einstein approach suffers from the absence of correlation effects, often resulting in an over-estimate for the ionic conductivity [61]. Whilst the Green-Kubo and Einstein-Helfand approaches can be used, it is also possible to correct the Nernst-Einstein equation using the distinct diffusion coefficients by [61]

18 $$\begin{aligned} \sigma ^{CNE} = \frac{q^{2}\beta }{3V}\Bigg (\sum _{\alpha }x_{\alpha }z^{2}_{\alpha }D_{\alpha } + \sum _{\beta \gamma }x_{\beta } x_{\gamma } z_{\beta } z_{\gamma } D_{\beta \gamma }\Bigg ), \end{aligned}$$

where the symbols are as above and the $D_{\beta \gamma }$ is the distinct diffusion coefficient for the $\beta , \gamma$ pair. MDSuite implements both the Einstein-Helfand and Green-Kubo approaches for the full ionic conductivity as well as the self and distinct diffusion coefficients thereby enabling the calculation of the Nernst-Einstein and corrected Nernst-Einstein conductivities.

#### Thermal conductivity

The thermal conductivity of a system describes the ability of a material to transport heat. At a macroscopic scale, thermal transport by means of conduction is described by Fourier’s law:

19 $$\begin{aligned} \textbf{q} = -\lambda \nabla T. \end{aligned}$$

In Eq. 19, $\lambda$ is defined as the thermal conductivity of the material and can be expressed using a Green-Kubo relation as:

20 $$\begin{aligned} \lambda = \frac{V}{3 k_\text {B} T^2} \int \limits _{0}^{\infty } \textrm{d}t \langle \textbf{J}^{\kappa }(0) \cdot \textbf{J}^{\kappa }(t) \rangle , \end{aligned}$$

where $\textbf{J}^{\kappa }$ is the heat-flux defined as:

21 $$\begin{aligned} \textbf{J}^{\kappa }(t) = \frac{1}{V} \Bigg [\sum _i e_i \textbf{v}_i(t) - \frac{1}{2}\sum _{i<j} \textbf{F}_{ij}(t) (\textbf{v}_i(t) +\textbf{v}_j(t)) \textbf{r}_{ij}(t) \Bigg ]. \end{aligned}$$

The equivalent Einstein-Helfand relation is also implemented for this quantity as

22 $$\begin{aligned} \kappa = \frac{1}{V k_\text {B} T^2} \lim _{t \rightarrow \infty } \frac{1}{2t} \langle |{\varvec{\mathcal {J}}}(t)- {\varvec{\mathcal {J}}}(0)|^{2} \rangle \end{aligned}$$

where ${\varvec{\mathcal {J}}}(t)$ is defined as the integrated heat current. This current can be stored during a simulation or computed in MDSuite as:

23 $$\begin{aligned} \varvec{{\varvec{\mathcal {J}}}}(t) = \sum \limits _{i=1}^{N} e_i(t) \cdot \textbf{r}_i(t), \end{aligned}$$

where $e_i(t)$ and $\textbf{r}_i(t)$ are the energy and the position vector of the *i*-th particle in the simulation respectively. In all cases, the per-particle energy must be printed during the simulation run in order for MDSuite to perform the computation. This can be done for example, using the LAMMPS [39, 40] compute pe/atom command which will dump the energy of an atom up to the specified cutoff including non-local terms such as electrostatics. In its current state, MDSuite makes no assumptions about inter-particle interactions.

### Transformations

In MDSuite, transformations are defined as operations on simulation data that yield time-dependent results. Because these properties exist at each time step, they are also stored under new groups in the MDS-DB.

Transformation may be called from a Project by:
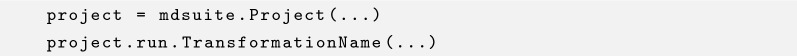


In the case where a transformation is required for a calculator to run, it will be called automatically by the MDSuite dependency handler. Whilst many of the transformations have already been discussed in the calculator section including ionic current, translational dipole moment, thermal flux, and the integrated heat current, MDSuite also offers some unique transformations which warrant additional discussion.

#### Coordinate (Un)wrapping

A core component of particle-based simulations is the use of periodic boundary conditions (PBC) to mimic a bulk system of infinite particles [47]. When post-processing is then performed on these systems, the application of PBC sometimes must be reversed in order to retrieve for example, correct dynamics, or to complete a molecular structure. In MDSuite, two approaches may be taken to unwrapping coordinates, box-hopping detection and scaling by image numbers stored during a simulation.

#### Molecule mapping

A notable transformation in MDSuite is the molecular mapping module. In this module, a distance search is used to perform graph decomposition on a configuration in order to map free particles into molecule groups. These groups can then be used with any of the aforementioned calculators thereby allowing for the construction and analysis of coarse-grained representations. To further improve the accuracy of this method, approximate graph isomorphism checks may be applied to ensure that the molecular graph built is approximately isomorphic with a reference graph constructed from the SMILES string (see Additional file 1). While molecule mapping module is very flexible and can be used to construct any number of molecule groups, it is currently hampered by computational limitations. In its current state, the initial construction of groups, usually performed on one or two configurations, is an $\mathcal {O}(N^{2})$ operation whereas the mapping of these groups over the full trajectory is $\mathcal {O}(N)$. Current work is underway in constructing more accurate and faster approaches to this molecular mapping.

### Customization

An important aspect of any post-processing tool is an ability to adapt its features for custom analysis. Through the use of object oriented programming, MDSuite provides users with the ability to subclass the parent calculator classes and in doing so, take full advantage of the database interface, memory safety, and performance. Due to the Python interpreter, these additions can be made without any reinstallation of the MDSuite software. Detailed information about this process is provided on the MDSuite developer documentation page at https://mdsuite.readthedocs.io/en/main/_developer_docs/implementing_calculators.html.

### Software and development

Aside from the features and performance of MDSuite, the development process is of utmost importance for stable and usable software. MDSuite utilizes a test-driven development approach where both unit tests and integration tests are used to cover the code base. Furthermore, continuous integration is used to ensure that before any code is added to the main branch the package installs for all supported Python versions, the documentation builds, and all of the tests and example scripts pass. MDSuite adopts the semantic versioning approach [63] and aims for a small number of major changes. MDSuite is released under the OSI approved Eclipse Public License 2.0 (EPLv2) and can be installed on Linux, Windows, and MacOS operating systems via pip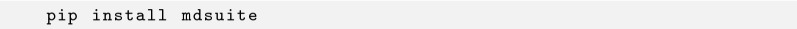 or directly from the github repository at https://github.com/zincware/MDSuite.

### Data visualization

Data visualization and exploration is of fundamental importance particularly in the age of big-data. In MDSuite, focus is placed on exploratory data analysis, that is, plots and visualizations that allow users to move through the data and identify or operate on regions of interest.

#### Three-dimensional visualization

**Fig. 6:**
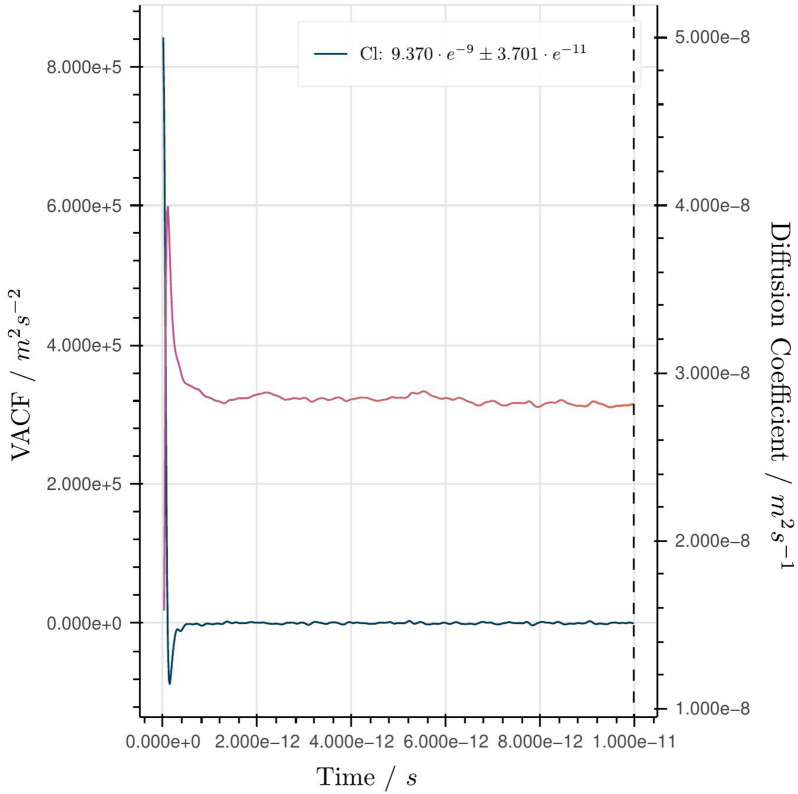
Interactive plot environment offered by Bokeh [65]. In this case, we see a velocity autocorrelation function along with the cumulative integral to identify a point of convergence

The ability to visualize a simulation or any particle trajectory can lead to better insights and intuition during analysis. MDSuite offers a rudimentary visualization module based on the ZnVis particle simulation visualizer []. ZnVis is built on top of the Open3D data processing engine [64] which utilizes a C++ backend for the visualization of point-cloud and mesh data. In MDSuite, ZnVis is used to display particles in an interactive window (Figs. 5 and 6), visualize the trajectory of small simulations, and capture snapshots of a simulation at a specific time step as a png file.

#### Two-dimensional visualization

In cases where a full three-dimensional visualization is not required, MDSuite utilizes the Bokeh [65] library to construct interactive plots for data analysis and exploration. These plots, displayed within a web-browser at the end of a calculation, are fully interactive in that they enable exploration of plotted data through zooming, sliding, and hovering over points in the plot. In addition to the automatic plotting features, user have complete access to the raw calculation data for custom plotting.

## Results and discussion

### Performance

**Fig. 7:**
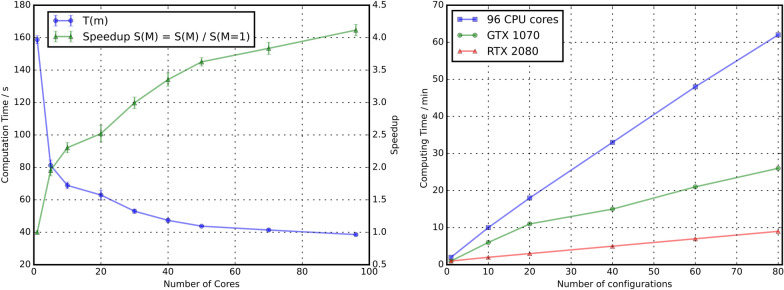
(left) Strong-scaling of the RDF calculation on 99000 Oxygen atoms. (right) GPU time scaling with respect to number of 108'000 argon atom configurations in an RDF computation

Beyond providing functionality, MDSuite has been developed to maximise the performance of computations. Much of the performance of the MDSuite calculators and transformations arises from the heavy use of TensorFlow [46] and the formulation of computations as tensor operations. By utilizing TensorFlow for these operations the computations can immediately be performed in parallel and on GPU with little to no additional software. This, coupled with the memory manager, results in memory safe and computationally efficient calculations. To assess the performance of MDSuite, a strong-scaling test has been performed using the RDF calculator as it presents both a memory and time intensive operation that is frequently used in computational studies. Strong-scaling measures the improvement in the time of a computation with respect to additional computational resources. In this study, the CPU access of TensorFlow was limited from 1 to 96 cores and the RDF computation performed 10 times to ensure correct statistics. In addition to the strong-scaling test, the calculations were also performed on several devices to assess performance improvements with respect to accelerators including a GTX 1070 and RTX 2080 GPU. In the strong-scaling test, 3 configurations of 33’000 oxygen atoms were used in the RDF computation. To additionally test the memory management capabilities of MDSuite, a 108’000 atom simulation of liquid argon was performed using the LAMMPS [39] simulation engine generating 5000 time-steps in the process , the trajectory of which was used in the device scaling tests.. In the device test, an RDF calculation has been performed on an increasing number of configurations, or frames, of this simulation. In the largest case (80 configurations), $80 \cdot 108000 = 8640000$ atoms are used in the RDF computation on devices with as little as 8 GB of memory. Fig. 7 outlines the results of these experiments. It is clear when studying the figure that the parallelization of the computation results in improved speeds.

In addition to plotting simply the computation time, the speedup factor is also included on the second y-axis of Fig. 7. This plot shows the factor speedup upon the introduction of additional resources and demonstrates how MDSuite natively scales to more capable computational devices. Turning attention now to the device scaling test, it can be seen that the deployment of the calculation onto a GPU results in a substantial improvement in computation speed over even the 96 core CPU computation. Furthermore, due to the use of the TensorFlow library [46] no specific GPU code was required for the acceleration. While the scaling of the MDSuite library is effective at improving computation times, it is not perfect scaling, i.e, with increasing resources vs computations the time plot will have a non-zero gradient. This may be partially addressed with continued optimization of the calculators, however, on some fundamental level this will always be limited by the performance of the TensorFlow library.

## Conclusion

We have introduced a new post-processing suite for particle-based simulations capable of combining the simulation data from several investigations, performing analysis on each, and comparing the outcomes of this analysis under a single framework. The Python library consists of an easy-to-use API thereby promoting accessibility to those in the community unfamiliar with programming.

Implemented methods of analysis combine libraries for tensor operations such as TensorFlow in order to optimize performance and provide better support for GPU and cluster deployment. Beyond a direct focus on performance, MDSuite also provides a memory-safe framework and in doing so, allows for the analysis of million atoms systems on desktop machines.

Whilst the current state of MDSuite is capable of a wide range of analysis, there is always more to be done. In future releases, development will focus on the extension of MDSuite calculators to other fields including colloidal studies, biological systems, and ideally, areas in high-energy physics.

The outlook of computational methods is promising. With the ever-increasing capabilities of interaction models and simulation engines, the possibility for new discoveries continues to grow. MDSuite offers a new, innovate means to combine scientific research with computational methods under a common framework.

## Supplementary Information

**Additional file 1. **  Additional information regarding molecule mapping, data processing, and memory management algorithms.

## Data Availability

Project name: MDSuite Project home page: https://github.com/zincware/MDSuite Operating system(s): Platform independent Programming language: Python3 License: EPL v2.0

## Abbreviations

PB: Particle based
MD: Molecular dynamics
MDS-DB: MDSuite database used for the storage of simulation data
SQL-DB: SQL database used by MDSuite to store the analysis of computations
RDF: Radial distribution function
ADF: Angular distribution function
KB: Kirkwood-Buff
PMF: Potential of mean-force
GPU: Graphics processing unit
CPU: Central processing unit
API: Application programming interface
PBC: Periodic boundary conditions
GK: Green-Kubo
EH: Einstein-Helfand
