# Supplementary material for: MDSuite: comprehensive post-processing tool for particle simulations
Source: J Cheminform. 2023 Feb 11;15:19. doi: 10.1186/s13321-023-00687-y (PMC9921696; doi:10.1186/s13321-023-00687-y)
Supplement: Supplementary file 1 — Additional file 1. Additional information regarding molecule mapping, data processing, and memory management algorithms. [file 13321_2023_687_MOESM1_ESM.pdf]

SOFTWARE

Supplementary information for MDSuite: comprehensive post-processing tool for particle simulations

Samuel Tovey[1], Fabian Zills[1], Francisco Torres-Herrador[2,3,4], Christoph Lohrmann[1], Marco Brückner[1] and Christian Holm[1]

1 Molecule Mapping

Molecule mapping is a crucial component to many simulation studies and the implementation of it in MDSuite offers a range of potential applications. In this section the process of molecule mapping is discussed in more detail as well as the isomorphism checks used to further ensure that the correct molecules have been detected.

1.1 Molecule interface and detection

The molecele mapping in MDSuite is dictated by the information the user provides the molecule graph module. This is interfaced through the `mdsuite.Molecule` class which contains information about the structure of the molecue, as well some system information such as how many should be in each configuration. An example of this class is shown below:

```
import mdsuite as mds

water = mds.Molecule(
            name="water",
            smiles="[H]O[H]",
            amount=200,
            cutoff=1.6,
            mol_pbc=True
)
```

Listing 1: An example of an MDSuite molecule for water.

Alternatively, for custom molecules where SMILES strings cannot be built, users can use a dictionary reference such as:

```
import mdsuite as mds

my_polymer = mds.Molecule(
            name="custom",
            species_dict="{"Group_1": 3, "Group_2": 2}",
            amount=10,
            cutoff=16,
)
```

Listing 2: An example of an MDSuite molecule for a custom molecule group.

At this stage in MDSuite, there is no difference between the use of SMILES and the direct use of a species dictionary. In the future, with the addition of even stricter isomorphism test, there will be some distinction in how exact the molecule mapping can be performed without a reference graph.

The actual mapping of the molecules takes place in the following steps:

1 Build distance tensor between all particles of the species contained in one molecule.

2 Apply mask built from cutoff value to construct adjacency matrix for all particles.

3 Perform graph decomposition on full adjacency matrix to collect matrices of individual molecules.

By the end of this stage, what is returned is a list of the different molecule on which certain isomorphism test can be performed to further ensure their validity.

1.2 Isomorphism tests

In order to try and validate that the chosen molecules are correct, MDSuite performs small isomorphism tests and raises an error if they fail. The tests performed are listed below along with why they are chosen and what a user can do in the event that they fail.

1 **Molecule amount test:** In this test, MDSuite simply checks whether the total number of molecules matches that of the reference information. This is the first and most simple test performed. Typically, a failure here is as a

result of an incorrect cutoff. If the cutoff is too large, not enough molecules are found, if it is too small, too many molecules will be found.

2 **Group equality test:** The second, and at this stage final, test performed by MDSuite is that of Group equality. When MDSuite constructs molecules it does so by building reference data structure with information about which particle species are in each molecule as well as how many. For example, for a water molecule it would state that there should exists one oxygen and two hydrogen particles in each molecule. This test checks to see whether this is true for all of the computed molecules. If this test fails, particles are either not being found, or are so close at this point in the simulation that the module cannot separate them. Resolving this failure is usually achieved by adjusting the cutoff value provided, or by using an alternative reference configuration in which the molecules are more separated.

2 Algorithms

Throughout the main article reference has been made to several algorithms employed by MDSuite for several calculations. In this section, we expand on why some of these approaches are used as well as where they can be found in the code.

2.1 Savitsky Golav Filter

In several types of analysis it is necessary to determine the minimum of a curve within a specified range. To perform such an analysis, it is important the curve being studied is smoothed, or at least, has a smoothed variant that can be used as a reference point for the true data. This point is addressed in MDSuite by using a Savitzky-Golay, or SavGol filter[1], to produce a smoothed copy of a function before a peak-finding code is applied. The filter works by expanding each data point out in a set of low-degree polynomial functions as

$$Y_i = \sum_{\frac{1-m}{2}}^{\frac{m-1}{2}} M_j y_{i+j}, \tag{1}$$

where M_j are convolution coefficients taken from an order n polynomial. In MDSuite, these parameters can be set by the user for the repsective application. The main benefit of SavGol filter compared to alternatives, such a moving average file, is that the location of the maxima and minima remains unaltered. In MDSuite,

the Scipy implementation of the SavGol filter is called within a separately defined method to apply the filter operation.[2].

2.2 Golden Section Search

Another algorithm implemented in associate with minimum detection is the Golden-Sector search algorithm developed by Kiefer in 1952[3]. The algorithm works as most extremum search methods do, by selecting points iteratively and reducing the search range until the value is found. In order to optimise the rate at which the algorithm works, successive intervals are chosen such that the new test range satisfies the golden ration. By providing a suitable range, the Golden-Sector search algorithm converges onto a function minimum with excellent accuracy. In order to ascertain an error value, the MDSuite implementation returns a range within which the minimum exists.

2.3 Min-Finding

The Golden-Section search algorithms is not enough on its own to ascertain the minimum value of a function as it requires and defined range over which to look for an extremum. Rather, in MDSuite, we combine the filtering capacity of the SavGol filter with the min-finding capabilities of the Golden-Section search. First, the SavGol filter is applied to the raw signal in order to generate a smoothed copy of the function. On this copy, the typical scipy peak-finding algorithm is applied which simply uses neighbour comparisons to detect the maximums of the function. These peaks are then parsed to the Golden-Section search algorithm as an initial range within which the minimum exists.

3 Memory Management

Memory management is a fundamental concept at the heart of MDSuite. The ability to perform memory safe analysis enables MDSuite calculators to examine the largest systems on almost any available device. Beyond providing a foundation for the analysis of large data-sets, memory safety also provides the means for pushing expensive calculations onto performance devices such as GPUs or, theoretically, TPUs.

MDSuite achieves memory safety by computing the scale functions of our calculators. This is to say, each calculator and transformation has with it an associated

scaling function which describes how the function scales with respect to additional particles or time-steps. At the beginning of each calculation, the theoretical memory consumption of a computation is derived and a batch size computed accordingly. If there exists no batch size capable of running the analysis, MDSuite will then move to so-called atom-wise or particle-wise batching. In this case, rather than trying to load in as many configurations as possible with all of the particles, this memory requirements are computed using a subset of the available particles. Therefore, the minimum memory requirement of any device is a single particles worth of the desired data range in the case of a dynamics calculation.

Author details

[1]Institute for Computational Physics, Universität Stuttgart, Stuttgart, Germany. [2]Aeronautics and Aerospace department, von Karman Institute for Fluid Dynamics, Rhode-St-Genese, Belgium. [3]Thermo and Fluid dynamics (FLOW), Vrije Universiteit Brussel, Brussels, Belgium. [4]Laboratory for Chemical Technology (LCT), Ghent University, Ghent, Belgium.

References

1. Savitzky, A., Golay, M.J.E.: Smoothing and Differentiation of Data by Simplified Least Squares Procedures. Analytical Chemistry **36**(8), 1627–1639 (1964). doi:10.1021/ac60214a047. _eprint: https://doi.org/10.1021/ac60214a047
2. Virtanen, P., Gommers, R., Oliphant, T.E., Haberland, M., Reddy, T., Cournapeau, D., Burovski, E., Peterson, P., Weckesser, W., Bright, J., van der Walt, S.J., Brett, M., Wilson, J., Millman, K.J., Mayorov, N., Nelson, A.R.J., Jones, E., Kern, R., Larson, E., Carey, C.J., Polat, I., Feng, Y., Moore, E.W., VanderPlas, J., Laxalde, D., Perktold, J., Cimrman, R., Henriksen, I., Quintero, E.A., Harris, C.R., Archibald, A.M., Ribeiro, A.H., Pedregosa, F., van Mulbregt, P., SciPy 1.0 Contributors: SciPy 1.0: Fundamental Algorithms for Scientific Computing in Python. Nature Methods **17**, 261–272 (2020). doi:10.1038/s41592-019-0686-2
3. Kiefer, J., Wolfowitz, J.: Stochastic Estimation of the Maximum of a Regression Function. Annals of Mathematical Statistics **23**(3), 462–466 (1952). doi:10.1214/aoms/1177729392
